# An iPPG-Based Device for Pervasive Monitoring of Multi-Dimensional Cardiovascular Hemodynamics

**DOI:** 10.3390/s21030872

**Published:** 2021-01-28

**Authors:** Jingjing Luo, Junjie Zhen, Peng Zhou, Wei Chen, Yuzhu Guo

**Affiliations:** 1 Institute of AI and Robotics, Academy for Engineering and Technology, Fudan University, Shanghai 200433, China; luojingjing@fudan.edu.cn; 2 Jihua Laboratory, Guangdong 528000, China; zpzp@tju.edu.cn; 3 School of Precision Instruments and Opto-Electronics Engineering, Tianjin University, Tianjin 300072, China; henri1745@tju.edu.cn; 4 Center for Intelligent Medical Electronics, School of Information Science and Technology, Fudan University, Shanghai 200433, China; w_chen@fudan.edu.cn; 5 Human Phenome Institute, Fudan University, Shanghai 200433, China; 6 School of Automation Science and Electrical Engineering, Beihang University, Beijing 100191, China

**Keywords:** photoplethysmography imaging, systematic design, cardiovascular activities, facial regional analysis, pervasive healthcare

## Abstract

Hemodynamic activities, as an essential measure of physiological and psychological characteristics, can be used for cardiovascular and cerebrovascular disease detection. Photoplethysmography imaging (iPPG) can be applied for such purposes with non-contact advances, however, most cardiovascular hemodynamics of iPPG systems are developed for laboratory research, which limits the application in pervasive healthcare. In this study, a video-based facial iPPG detecting equipment was devised to provide multi-dimensional spatiotemporal hemodynamic pulsations for applications with high portability and self-monitoring requirements. A series of algorithms have also been developed for physiological indices such as heart rate and breath rate extraction, facial region analysis, and visualization of hemodynamic pulsation distribution. Results showed that the new device can provide a reliable measurement of a rich range of cardiovascular hemodynamics. Combined with the advanced computing techniques, the new non-contact iPPG system provides a promising solution for user-friendly pervasive healthcare.

## 1. Introduction

Pervasive monitoring of cardiovascular health is attractive for both clinical and biomedical communities [1]. However, existing monitoring equipment is either embedded in medical institutes or requires a specialist for operation. There is a shortage of user-friendly cardiovascular monitoring equipment which is informative with easy accessibility, allowing various health indices to be detected without knowing the detailed underlying mechanisms [2,3] for daily life healthcare. iPPG (Photoplethysmography Imaging) which is based on a non-contact and imaging technique [4,5,6], offers the public a potential solution for quick and smart self-monitoring, and is being increasingly used in such applications.

iPPG measurement is often based on contact and point-light plethysmography (PPG), where compression of contact tissue is essential and can only be measured at specific body regions. The non-contact technology provides unconstrained blood volume pulse measurements for cardiovascular or microcirculation monitoring. Several physiological information, such as the blood oxygen saturation calculation [7], pulse transit time (PTT) [8], heart rate (HR) and its variability (HRV), can be obtained through analyzing of iPPG sig dxnals [9]. HRV derived in certain regions can be used to analyze the function of parasympathetic nervous system [10]. PTT reflects the changes in cardiovascular parameters, such as arterial elasticity and stiffness [8,11], which can be derived from the spatial dynamic analysis of iPPG with pulsation distribution

Additionally, iPPG can provide extensive information on the vascular network under the skin by measuring the spatial distribution of dynamic pulsations; thus, multi-dimensional biophysical details of physiological parameters can be derived for analyzing cardiovascular functions. For example, the spatiotemporal information derived from iPPG measurement can be used to detect blood perfusion distribution at irritated skin [12] and symmetric facial distribution in potential migraine patients with family history [13]. High-dimensional information from iPPG has also increasingly been used for human–machine interaction in healthcare applications. The radial artery above radius changes pulsatory volume has been localized using the video-based iPPG technique [14].

Despite increasing potential applications, the essential iPPG equipment for healthcare monitoring service has rarely been reported. iPPG-based healthcare equipment consists of a lighting source, a camera, and iPPG derivation algorithms. Tulyakov et al. [15] extracted iPPG, using ambient light, from regions of interest (ROI) selections, excluded from independent movement’s influence. Kwon et al. [16], using visible lighting in an office environment, studied the division of face ROI regions based on the signal-to-noise ratio (SNR). Hao-Yu et al. [17] used a web-camera to take videos in ambient light and proposed a method to enhance the subtle intensity changes in pixel values reflecting facial blood pulsation. Kamshilin et al. [18] used the green Light Emitting Diode (LED) equipped with a Complementary Metal Oxide Semiconductor (CMOS) camera to calculate PTT and Blood Pulse Amplitude (BPA) distributions from subjects in sedentary and recumbent positions. However, most current iPPG applications were set up in laboratory environment [19,20].

In this study, a new multi-functional iPPG sensoring device, integrated with signal processing algorithms, was proposed for monitoring high-dimensional spatiotemporal pulsation information. The system defines hardware configuration of the green-lighted camera-based iPPG system and ROI selection criteria. iPPG spatial distribution was visualized to demonstrate the feasibility of facial hemodynamics analysis. The highly integrated system adds convenience and flexibility for the user, and allows an interactive self-monitoring of multi-dimensional physiological information at home or for public health applications

## 2. Materials and Methods

### 2.1. The iPPG Genesis of The Device

**Figure 1:**
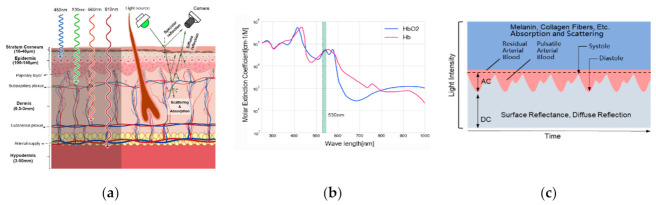
(**a**) Illustration of incident light penetration of different wavelengths through layers of the skin and skin reflection mode. In these wavelength ranges, a longer wavelength represents a higher light penetration rate. Green LED at 530 nm can reach the subpapillary plexus of the dermis layer; (**b**) Absorption spectra of deoxygenated hemoglobin (Hb), and oxygenated hemoglobin (HbO2). The x-axis represents the wavelength, and the y-axis represents the absorption rate. A local maxima absorption rate at 530 nm (green bar); (**c**) The iPPG’s AC and DC formation from light intensity rely on the light reflection and the arterial blood flow over time.

The dynamic fluctuations of reflection of incident light from dermal surfaces are recorded by optical sensors (Figure 1a). A green LED with a wavelength of 530 nm was used. The estimated depth of penetration (where the illuminance is attenuated by 95%) is ~1 mm [21,22], where lies the subpapillary plexus layer. Furthermore, the molar extinction coefficient of hemoglobin reaches its locally maximized ~530 nm (Figure 1b), thus is optimized for the detection of pulsation volume changes [23]. According to the Lambert–Beer law and scattering theory, body tissues, such as bones, muscles, venous blood, and melanin, absorb constant amounts of light, which constitutes the direct component (DC) of iPPG. Changes in arterial blood volume in each cardiac cycle [24] produce the alternating component (AC) (Figure 1c).

### 2.2. Devise Composition

**Figure 2:**
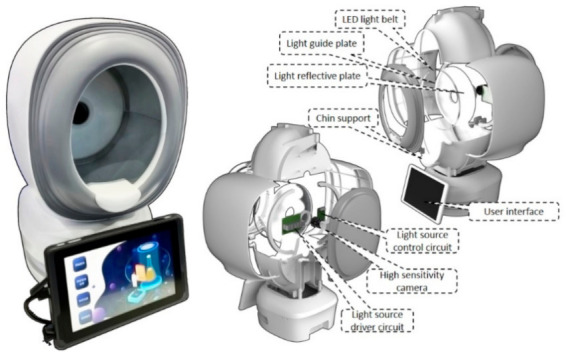
The composition of equipment. Left is the picture of the equipment, including the illumination chamber and a Tablet PC for the user interface (UI). Center picture shows the light-source driver, controller, and camera, all of which have been fixed at the back of the chamber. Right shows the light source’s composition, including the light guide, scattering, and reflective plates. The LED-belt light source is around the rim of the light guide plate. Users can place their chin on the chin support and start video recording and iPPG analysis.

The new device comprises a lighting system, an enclosed illumination chamber, a recording camera and a user interface. iPPG is measured within an enclosed environment illuminated with designed light sources to avoid environmental light interferences (Figure 2). A round LED belt light with adjustable intensity (via USB 3.0) was used, composed of light sources of the green light with a wavelength of 530 nm. It was ensembled with a light guide, and reflective and scatter plates to ensure uniform light emitting outward, placed at the equipment chamber’s deep end. The illumination chamber has an elliptical opening of a size 15 cm × 20 cm to fit an entire human face. Chin support was set 25 cm away from the camera to help fix head posture and avoid head movement.

A high sensitivity camera of Basler^TM^ aca2440-75ucmed camera (chip type: CMOS, highest resolution of 2448 px times 2048 px, maximum frame rate: 100 fps, data recorded: 8 bit, manufactured in Ahrensburg, Germany) is installed. It is arranged in the middle of the plate, equipped with a low-distortion c-mount 12 mm lens (m0814-mp2) to capture image sequence under light illumination. An interactive user software was developed integrated within a touch screen for customers to operate the device.

### 2.3. Subjects

In this study, a total number of 9 volunteers were tested, aged between 22 and 36 (25.9 ± 4.9) and without a history of critical illness. The volunteers’ blood pressure was recorded with Omron^TM^ HBP-9021 (manufactured in Dalian, China) before the experiment, along with general health information. Participation was voluntary, and all participants gave written informed consent.

### 2.4. Experimental Setup

**Figure 3:**
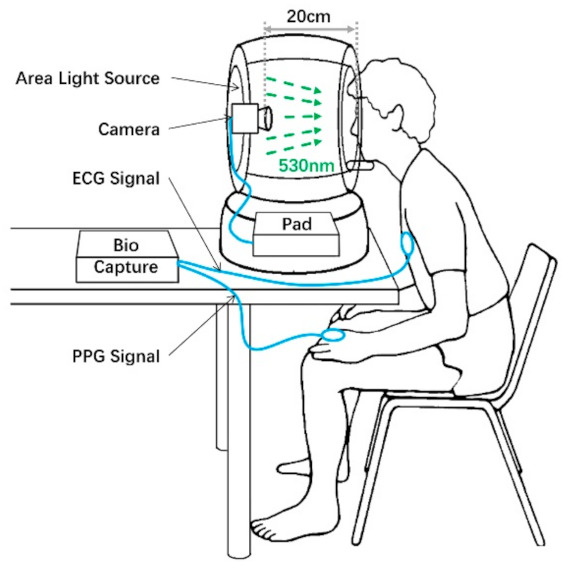
Experimental setup to record iPPG, PPG and ECG data. Biocapture ^TM^ was attached to the left hand’s index finger for PPG signal detection and to the chest for ECG signal detection. Meanwhile, our equipment records facial video with the green light on.

In an experiment, the volunteer was set in a calm position, and the distance between the face and the camera is around 20 cm. The camera’s exposure time was set at 20,000 µs, and the frame rate was 40 fps. The size of each frame was 1800 px wide and 2000 px high. We achieved the illumination at face as 850 lx. A 30-s sequence of images is continuously recorded. During the experiment, BioCapture^TM^ (manufactured in Cleveland, U.S.) was also used to collect finger PPG and electrocardiogram (ECG) signals for reference, illustrated in Figure 3.

### 2.5. Analysis Methods

#### 2.5.1. Facial Region Segmentation Based on Facial Landmarks

The present study applied the ROI segmentation [11,12,13,14,15,16,17,18] process into three parts: the face detecting process, the facial landmarks detecting process and the region’s auto-generation process. We used Dlib toolkit’s [25] to detect the face and “shape-predictor-68-face-landmarks” to find the facial landmarks. As the face detection algorithm returns rectangle areas for preliminary facial location, an 81-facial-landmarks algorithm retrained from [26] returns coordinates of facial locating points within the rectangle region. According to the combination of the locating points, we defined 98 (0~97) non-overlapping, seamless, and symmetry triangle regions to cover the whole face. Then, the fixed-size rectangles (80 px × 80 px) were applied in each region’s centroids, and the regional iPPG signals were extracted from these rectangles (ROI). Considering the relatively static face position and requirement of real-time iPPG signal processing, we built a ROI mask for the first image. We used it to extract the corresponding regions from the follow-up input pictures.

#### 2.5.2. Signal Processing

We used the green channel of images. Pixels within the selected ROI were averaged to reduce spatially uncorrelated noise and derive “raw iPPG signal” following a multi-step iPPG extraction procedure [12]. Prior to further analysis, the low frequent component was removed by mean-centralization (also called sliding mean) using a sliding 1s window. To extract “pre-processed iPPG signal”, a 0.5–4 Hz bandpass filter using 5^th^ Butterworth was applied to derive the. A 0.2–0.5 Hz bandpass filter using 5^th^ Butterworth was applied to derive the “breathing signal”.

#### 2.5.3. Q Value for Evaluating Signal Quality

SNR is derived from the power difference between pre-processed iPPG and raw iPPG, using the equation below:

(1) $$SNR=10\times \log_{10}(\frac{\sum_{i}^{N}f{\left(i\right)}^{2}}{\sum_{i}^{N}{\left(f\left(i\right)-x\left(i\right)\right)}^{2}})$$

where *x(i*) is the *i*^th^ sampled value of raw iPPG signal, *f(i)* stands for the *i*^th^ sampled value of signal processed by the bandpass filter.

To evaluating the signal quality, standard deviation of AC (denoted as STD), and SNR were used to define a *Q* value as:

(2) $$Q=mean\;\left(SNR\right)+mean\left(-STD\right)=\overset{N}{\underset{n-1}{\sum }}\left(SNR_{n}-STD_{n}\right)/N$$

where *N* is the number of the subjects. Further process of iPPG analysis was applied after sorting by *Q* value and within the chosen top 50 regions.

#### 2.5.4. Time Lag

The 20th region (the middle of the forehead) was selected as the reference region for analysis of the average time lag difference of the top 50 ROI regions selected. We calculated the peak arriving time difference between the reference area and other regions to measure time lag:

(3) $$T_{i}=\overset{K}{\underset{k=1}{\sum }}\frac{P_{i,k}-P_{ref,k}}{fps}$$

where *P_i,k_* is the *k*^th^ peak point of region *i*, *P_ref,k_* is the *k*^th^ peak point of the reference region, and k is the number of cycles.

#### 2.5.5. Mean of Difference

We define mean of difference as the time lag effect size that we are interested in. By applying bias-corrected and accelerated bootstrap resampling [27], 5000 samples were derived from each region’s data. Wilcoxon signed-rank test was used to measure the relationship between a pair of regions. By considering 95% is widely acceptable as confidence interval, *p*-value were calculated with 5% chosen as the threshold to reject the null hypothesis.

#### 2.5.6. iPPG Intensity

AC was calculated using pre-processed iPPG by calculating peak-bottom amplitudes. The equation below is used to compute the signal intensity of the recording period:

(4) $$AC={\left(\frac{\sum_{i=0}^{N}s{\left(i\right)}^{2}}{N}\right)}^{\frac{1}{2}}$$

where *s*(*i*) stands for the *i*^th^ sampled value of pre-processed iPPG signal and *N* stands for the number of sampled points in one period.

#### 2.5.7. Goodness Matrix

To estimate if a signal mainly consists of target frequency, M. Kumar et al. propose a metric called “Goodness Matrix” [28]. Goodness define as follow:

(5) $$G\left(PR\right)=\frac{\int_{PR-b}^{PR+b}Y\left(f\right)df}{\int_{B1}^{B2}Y\left(f\right)df-\int_{PR-b}^{PR+b}Y\left(f\right)df}$$

where *G* is goodness, *Y* is the power spectrum density of iPGG signal, *PR* is the periodical pulsation frequency, *b* is the frequency threshold, *B*1 and *B*2 is the frequency bandwidth of the iPGG signal. This goodness matrix is to quantify the iPPG signal quality around the pulse rate for each ROI, and *G* below 50% was excluded for intensity analysis. By multiplying *G* to *AC*, the weighted results can be obtained to highlight the pulse signal in the facial distribution analysis.

#### 2.5.8. Eulerian Video Magnification

Eulerian Video Magnification (EVM) is a method to reveal the temporal variation within a video proposed by Wu et al. [29]. EVM decomposes the video into pyramidal ranks of resolutions then applies temporal filtering to select a preferred frequency for emphasizing and amplification. Finally, ranked videos are summed back to reconstruct the composed signals and generate the magnified video. The reconstruction result is used to show the propagation and strength distribution of the pulsations clearly in the current work.

## 3. Results and Applications

### 3.1. iPPG Processing

**Figure 4:**
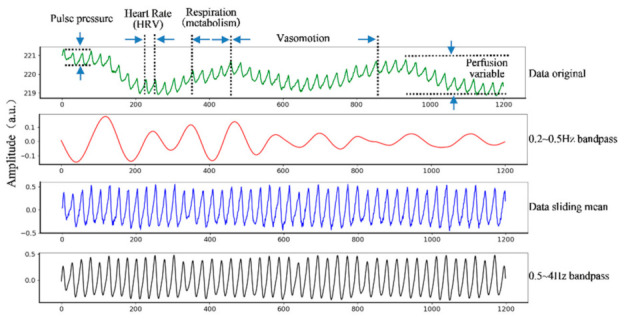
Original iPPG signal of recording. Multiple biophysiological indices, such as pulse pressure, heart rate variability, breathing rate, perfusion variable information can be derived from the signal. The x-axis is the recorded time frame, and the y-axis is the amplitude. The green line is the change of the original pixel value extracted from the picture. The red line is the breath signal after a 0.2~0.5 Hz bandpass. The blue line was after a 1s window sliding-mean process, and the black line is the smooth signal obtained after passing through a 0.5~5 Hz bandpass filter.

The raw iPPG signal derived from the non-contact images of the forehead can be used to derive multiple biophysiological indices (illustrated in Figure 4). A breathing waveform can be obtained by bandpass filtering the raw signals with the bandpass 0.2~0.5 Hz. The sliding mean process with 1-s window derived the mean-centralized iPPG signal. A 0.5–4 Hz bandpass filter using 5th Butterworth was applied to derive the “pre-processed iPPG signal”.

### 3.2. Regional iPPG Derivation

**Figure 5:**
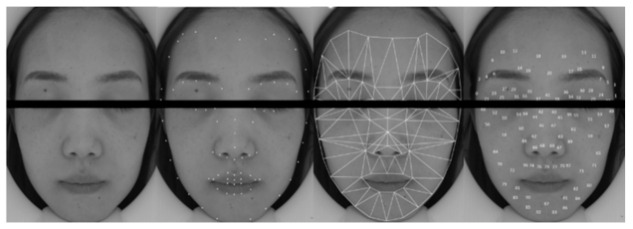
Facial landmarks detection and ROI extraction. Left two are the original image taken by the camera and the 81 facial features detected. Right two represent the 98 triangular regions formed by combinations of different landmarks and the number of each triangular region.

To analyze the differences between regions, this study derived regional iPPG for each subject by segmenting the facial areas using its geographical features (Figure 5). A total of 81 facial features were detected from each face image, constructing 98 triangular regions by combinations of neighboring landmarks. We recorded 36 groups of iPPG signals from 9 subjects (each subject tested four times in both resting state and post-exercise state), then sorted 98 facial regions according to the Q value of Equation (3) at each state. The deviation of the mean “-STD” is relatively lower in the higher-ranking regions, indicating the AC volatility tend to be low consistently for different subjects in these regions. Mean SNR and mean “-STD” were calculated as 11.96 and −0.08, respectively.

**Figure 6:**
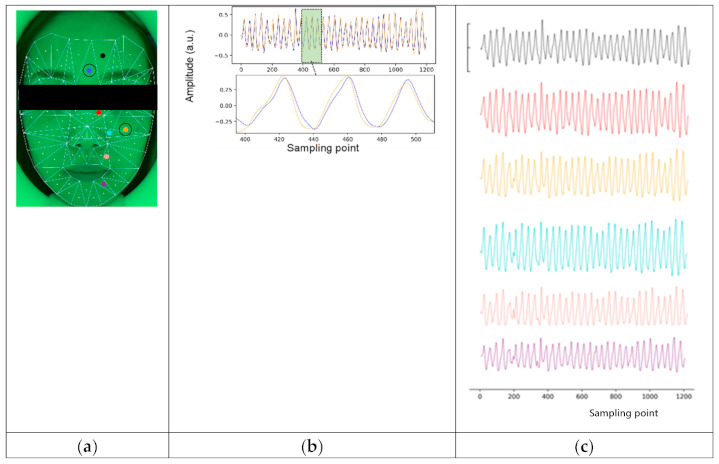
(**a**) Illustration of face region segmentation. The entire face is covered by a set of 98 triangles defined by facial landmarks combinations and each triangle has a dot indicating the location of the center of 80 px × 80px rectangles where the iPPG signal was extracted from; (**b**) Aligned waveform comparison of signal from meta area 20 and meta area 59; (**c**) Examples of iPPG signal from different meta regions (19,45,59,89,98,92). X-axis represents the sample points (40 Hz), y-axis represents the intensity of signal.

To explore differences between areas and face sides (left or right), we selected 38 symmetric meta-regions from the top 50 ranked by the Q value for further analysis, hereafter referred to as “meta area” or “meta region”. They formed a total of 12 larger symmetric areas (6 regions on each side), referred to as ”region” or “area” in Table 1. For illustration, we draw the waveforms of six meta regions (from meta regions 19, 45, 89, 98, and 92), and the comparison of waveforms of meta-region 20 and 59, as it’s shown in Figure 6. Waveforms in the selected region are rhythmic showing obvious peaks, and it can be found that peak times vary among these meta regions. This idea is the foundation of the time lag analysis between regions in the next step.

### 3.3. Time Lag Analysis

#### 3.3.1. Analysis among Vertical and Symmetrical Regions (ANOVA)

We used time lag, with meta area 20 in the middle of the forehead as reference, to measure phase difference among regions. Symmetrical areas composed in Table 1 were the left and right forehead, the left and right nose bridge, the left and right mid cheek, the left and right nasolabial fold, the left and right peri-oral, and the left and right chin areas. For each repeated experiment, we used Equation (4) to calculate time lag for 38 meta regions and obtained in total of 18 samples for each region.

One-way ANOVA was used to analyze the influence of region factors on both sides, and the statistical data were shown in first two rows of Table 1. The *p*-value the region factors of the left and right faces is 9.494 × 10^−5^ and 9.122 × 10^−13^, respectively, which are both less than 0.01. Therefore, it is considered that the categorical variable of the region has a significant influence on the regions’ time lag of both the left and right faces.

Two-way ANOVA was used to analyze the two regions and face sides and their interactions. The statistical data obtained were shown in the last three rows of Table 1. The results show that the *p*-values of side factor and region factor are 2.250 × 10^−3^ and 1.120 × 10^−17^, respectively, which are both smaller than 0.01. Therefore, it is believed that there is a significant difference in the time lag between regions in the whole face range, and there is a significant difference in the mean of the phase difference between the left face and the right face.

The *p*-value of the interaction of the two factors is 0.0647, as greater than 0.05, thus it is believed that one factor will not affect other factor’s effect on the peak arriving time, that is, the time lag between regions do not depend on whether regions come from the left cheek or the right cheek.

In conclusion, there are significant differences between regions, as well as between the left and right faces, but the regional differences are enough for us to ignore the differences between the left and right facial sides.

#### 3.3.2. Mean-of-Difference Analysis of Vertical Regions

To specify the difference among regions, we analyzed time lags for all regions with scatter diagram illustrating “time lag” and distribution diagram illustrating “mean-of-difference” with reference to the forehead meta region. A global trend of time lag changing from both sides of the face is shown: it went down from forehead to nose bridge and mid cheek, then ascended from mid cheek to nasolabial fold to upper peri-oral and to chin regions (upper scatter diagrams in both Figure 6a,b).

**Figure 7:**
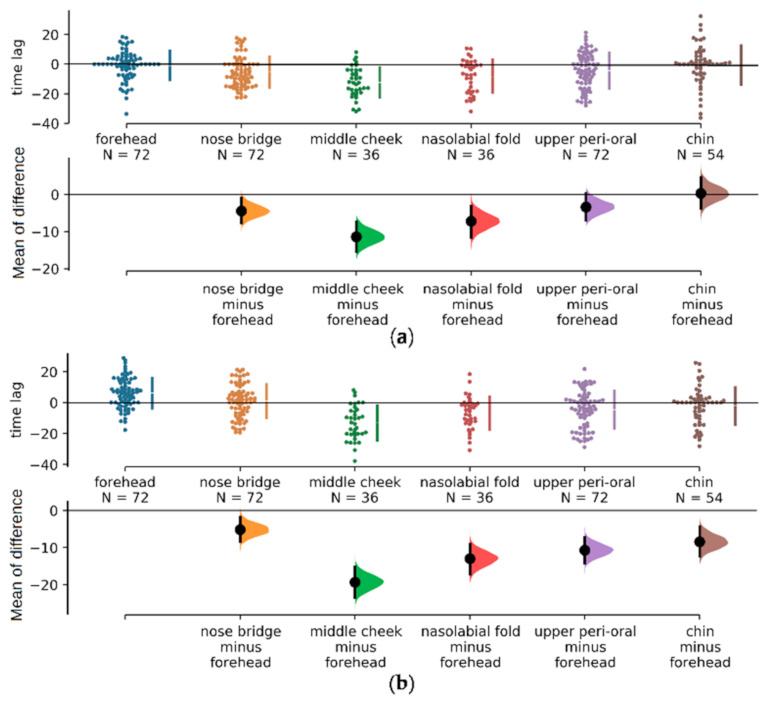
Comparison of time difference between each region and reference region. (**a**) Left face’s data demonstrated; (**b**) Right face’s data demonstrated. In the upper part of (**a**,**b**) is the scatter diagram of the time difference between the six regions (each region is composed of the meta region shown in Tab 1 and each meta-region has 18 sample points.) and the reference region. In the lower part of is the distribution of the time difference between the other five regions and forehead. The vertical axis is the time difference (ms), which is used to show the range of the 95% CI of the mean-of-difference. 5000 samples from BCa bootstrap were taken from the six regions of the left and right faces, and the forehead was taken as reference to calculate the probability distribution of difference between other areas and forehead, and we had a bar with a circle indicated the range of 95% CI of difference mean.

We also compared time lag between forehead and other regions by calculating “mean-of-difference”, shown in lower diagrams in both Figure 7a,b. We found that on the left side of face, the bars of chin and peri-oral region had crossed the zero baseline, in other words, there’s no significant difference between these two regions’ time lag from the forehead. As to regions of the right side, all null hypothesis was rejected, which means all regions were significantly different from forehead region.

#### 3.3.3. Mean-of-difference Analysis of Symmetric Regions

To analyze time difference between symmetric regions of left and right face sides, we compared and analyzed their time lags from Table 2. The maximum time difference between two symmetric areas was found in chin as −0.620 − (−2.245) = 1.625 ms, and the smallest was found in upper peri-oral as −4.291 − (− 4.492) = 0.201 ms. Time difference for forehead and nose bridge were significant, at −7.090 ms and −6.381 ms respectively.

**Figure 8:**
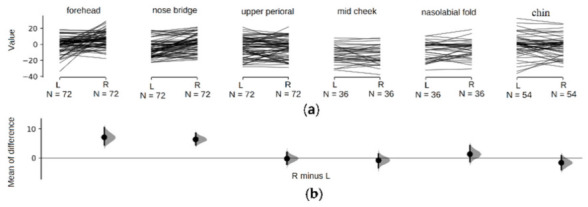
The phase comparison of the symmetrical regions of the left and right faces; (**a**) shows lines between the sample points of the corresponding region of the left and right face sides. The horizontal axis is the corresponding region, and the vertical axis is the time difference between the sample point and the reference point. (**b**) is the distribution map of the difference between the corresponding regions of the left and right faces. The horizontal axis marks the subtracting regions, and the vertical axis represents the time mean-of-difference between regions.

The diagram of time lag comparison and mean-of-difference between samples from symmetric regions were analyzed and shown in Figure 8a,b. There is significantly positive mean-of-difference between right and left in forehead and nose bridge regions. The mean-of-difference of the other areas is around zero (the zero baseline has crossed the 95% CI areas). As the *p*-value in Table 2 has shown, upper peri-oral, mid cheek, nasolabial fold and chin don’t have significant difference between left and right.

#### 3.3.4. Distribution of Pulse Amplitude and Time Lags

**Figure 9:**
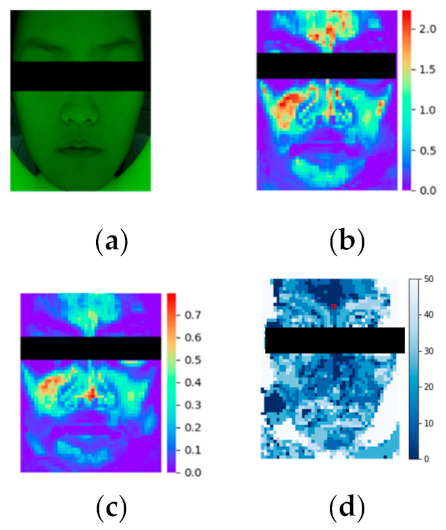
iPPG imaging analysis. (**a**) the original image, candidate’s 1of a period the video after EVM process (above row), and the bottom row is the iPPG waveforms of right-cheek (blue) and forehead ( frame image under 530nm’s green light; (**b**) the Goodness Matrix; (**c**) image of weighted AC, red means large-amplitude and represents the region with strong pulse-rate intensity; blue means small-amplitude and represents the region with weak pulse-rate information;(**d**) Time lag between forehead’s iPGG signal and each selected ROI’s iPGG signal.

We applied imaging analysis for intuitively observing the difference between iPPG intensity and time lags over face distributions. From the original facial video, Figure 9a as the 1st frame, the heatmap of Goodness matrix was calculated, shown as Figure 9b. The color distribution illustrates the area outside of the face not affected by pulse rate. The influence of heart-rate can be clearly distinguished by the red and yellow marks. iPPG intensity was calculated as the weighted AC image (Figure 9c), which demonstrated the effective intensity distribution of the face. The forehead and cheek had large amplitude, while the lower jaw has relatively lower. Imaging analysis of other subjects showed that areas composed of mainly heart rate signals were cheeks, forehead and jaws. Time-lag distribution was calculated as Figure 9d, demonstrating that forehead, nose bridge and chin have similar peak times. However, cheeks significantly increased in time lag in comparison. The results of visualization were consistent with results from statistical analysis.

#### 3.3.5. iPPG Enhancement and Visualization

**Figure 10:**
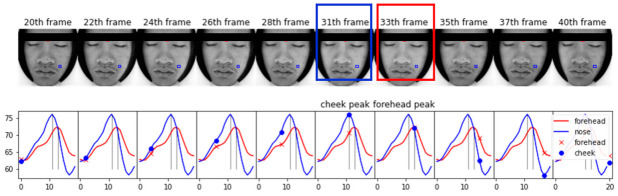
Illustration of EVM enhancement and visualized using video frames and iPPG waveform. This figure demonstrates 10 frames of a period the video after EVM process (above row), and the bottom row is the iPPG waveforms of right-cheek (blue) and forehead (red) ROI. Two vertical black line shows the gap of two peaks, cheek reach peak at 31th frame and forehead at 33th as expected.

To intuitively observe time lags between forehead and cheek, we can visualize the iPPG time lag from one period of a video sequence from a subject with a relatively slow heart-rate. Usually, it is difficult for the naked eye and the camera to distinguish heart rate change information; however, changes of facial color amplitude and brightness after EVM and be directly visualized, with pixel intensity of the face’s ROI changes periodically according to the heart rate. The brightness changed significantly in Figure 10, and pulse rhythm after EVM was illustrated below the iPPG waveform. Forehead’s peak time occurred at 33th frame, and the cheek’s peak occurred at 31^th^ frame; thus there is a time difference. The forehead reached a peak later than the cheek, as similar to statistical analysis.

## 4. Discussion

In this study, we introduced a user-friendly equipment for facial video recording, and our system showed strong robustness in iPPG extraction and pulsation distribution for high-dimension spatiotemporal applications. Typical physiological indices can be derived using multi-step iPPG processing. Facial regional analysis provides time lags between meta-region and the reference region by averaging the peak arrived time differences. ANOVA analysis demonstrated the significant time lag between forehead and cheeks while left-right regions are symmetric. Associated with video amplification algorithms, we also used this device to deliver visualization characteristics of the microcirculatory system, and subtle differences between different regions for potential applications of cardiovascular disease diagnosis.

Previous studies on facial region signal mainly focused on signal intensity or pulsation amplitude. In research [30], it suggested that watching a comedy movie increased the cheeks’ blood flows significantly. In [31], it discovered that painful tooth stimulation could induce temporal vasodilatation on bilateral cheeks. In this study, the signal’s physiological characteristics were explored from the time lag of signal waveform rather than just amplitude. This work reveals that the iPPG signals arrive in bilateral mid cheeks earlier than in other regions’ generally; in other words, this means the cardiovascular activities in bilateral mid cheeks have less delay than those of other regions.

The cause of facial distribution of iPPG intensity remains to be disclosed. [14] suggested that migraine is associated with lateralization of blood perfusion and asynchronous blood pulsations in the facial area. In [32], the partial correlation between facial regions’ signals and cold pattern questions was studied, and suggested that certain diseases would lead to a specific facial imaging pattern. In [28], researchers suggested that blood flows change for people with different emotions. In [33], it was proposed to match the spatiotemporal patterns of facial cardiovascular activities with cardiovascular diseases by decomposing regional iPPG signals into independent components and analysing their phase spectrum. There is a physiologically and psychologically diagnostic significance of studying the inter-regional difference and symmetry of iPPG patterns.

## 5. Conclusions

A multi-functional non-contact device has been developed to measure physiological signals based on the facial video. It is designed to be applicable pervasively while allowing for high quality iPPG extraction. A wide range of applications can be implemented, including multiple physiological indices, hemodynamic patterns and time lags from iPPG multi-step derivation or enhancement.

Integrated with advanced analysis algorithms, results showed that the new system was sensitive, rapid, and smart in cardiovascular hemodynamics screening. The device and the visualization processing provide a new option for monitoring vascular dynamic characteristics and allow intelligent health screening in both home circumstance and public healthcare applications.

## Figures and Tables

**Table 1:** ANOVA statistics of time lags at face regions.

Factor	DF-fac	DF-err	SSF	SSE	F-Value	*p*-Value
Within left face	18	323	6436	39810	2.894	9.494 × 10^−5^
Within right face	18	323	13333	39019	6.132	9.122 × 10^−13^
Between two sides	1	646	1148	78929	1.56	2.250 × 10^−3^
Between regions	18	646	16338	78930	7.429	1.120 × 10^−17^
Face sides × regions	18	646	3431	78929	1.56	0.0647

DF-fac stands for “degree of freedom of factor”, DF-err stands for “degree of freedom of error”, SSF stands for “sum or square for factor”. SSE stands for “sum of square for error”, and MSF = SSF/DF-fac, MSE = SSE/DF-err, F-value = MSF/MSE and it determines the *p*-value. DF-fac for regions (the 1st, 2nd and 4th rows of Table 1) is 19 − 1 = 18 (19 is the number of regions), DF for face side (the 3rd row of Table 1) is 2 − 1 = 1 (2 means “left or right side of face”), DF for interaction of two factors (the 5th row) is (19 − 1) × (2 − 1) = 18.

**Table 2:** Time difference between the face region and reference region.

Factor	DF-err	SSE	F-value	*p*-Value
forehead	−0.9 ± 9.8	6.2 ± 9.8	−7.1 ± 13.0	2.7 × 10^−5^
nose bridge	−5.3 ± 10.3	1.0 ± 10.8	−6.4 ± 8.7	3.1 × 10^7^
middle cheek	−12.3 ± 10.1	−13.1 ± 11.0	0.798 ± 7.2	0.640
nasolabial fold	−8.1 ± 11	−6.8 ± 10.5	−1.3 ± 8.3	0.633
upper peri-oral	−4.3 ± 11	−4.5 ± 12.0	0.2 ± 8.95	0.516
chin	−0.6 ± 13	−2.2 ± 12.1	1.6 ± 9.3	0.257
